# ‘Standing on the Shoulders of the Giants’: Dr. Giampaolo Merlini

**DOI:** 10.1007/s44228-023-00039-5

**Published:** 2023-03-20

**Authors:** Mohamad Mohty, Giampaolo Merlini

**Affiliations:** 1 Sorbonne University, AP-HP, INSERM UMRs938, Paris, France; 2 Service d’Hématologie Clinique et de Thérapie Cellulaire, Hôpital Saint Antoine, AP-HP, Paris, France; 3 Amyloidosis Research and Treatment Center, Fondazione IRCCS Policlinico San Matteo and University of Pavia, Pavia, Italy

In a letter to Robert Hooke in 1675, Sir Isaac Newton made his most famous statement: *“if I have seen further, it is by standing on the shoulders of giants”*

This initiative of the international academy for clinical hematology (IACH) aims to celebrate the achievements of leading experts and investigators whose work and research have helped to significantly advance the field of clinical hematology and establish the milestones and foundations of modern clinical hematology. This report represents a transcript of the interview given by Dr. Giampaolo Merlini (GM) on the 26th of July 2022 who responded to a series of questions asked by Dr. Mohamad Mohty (MM).

MM: Our honouree today is Professor Giampaolo Merlini from Italy, who has dedicated his career to amyloidosis. His contribution, his research and work have allowed the modification of the natural history of this terrible disease. Today, thanks to the work, effort and energy of Dr. Merlini, a significant proportion of these patients are cured or can live in complete remission for several decades. Dr. Merlini, welcome and thank you for joining us today for this special broadcast of the ‘Standing on the shoulders of the giants’. I would like to start by asking if you can tell us something about your early educational experience and how that might have played a role in your selection of scientific and medical fields.

GM: Thank you so much Dr. Mohty for this honour. I grew up in a small country village near Cremona, the birthplace of Stradivari and Monteverdi. In my early childhood I was influenced by an uncle, uncle Julio, who introduced me to the wonders of nature on long walks in the countryside, He instilled in me the curiosity to understand natural phenomena. In high school I became really fascinated by chemistry. So, when I had to choose a university, I was very undecided between chemistry and medicine. An old Professor of Chemistry suggested to me medicine and recommended me to focus on biochemistry. So, he told me that this way, you could have chemistry and also the biological aspects of chemistry. I will say that biochemistry was the basis of all my work in medicine and I would also like to add that I had the possibility, many years later to study biochemistry of proteins in Professor Jan Waldenström’s lab in Sweden, which was the key to all my subsequent research.

MM: So, there were several teachers and faculties that you thought were exemplary and have influenced your career, am I correct?

GM: Yes Dr. Mohty, you are right. My academic mentor in the Faculty of Medicine at the University of Pavia was Professor Sergio Perugini who was part of the great Italian hematology school, fathered by Adolfo Ferrata. Professor Perugini showed me the importance of traditional research, the advantage of standing on the shoulder of a master and the obligation, at the same time, to pass on to future generations a field with the deeper knowledge of the disease and better care thanks to translational research. After my graduation, my two main mentors were Jan Waldenström in Sweden and Elliott Osserman at Columbia University, New York.

MM: Well, that brings me to an important question, as you may guess, what attracted you in particular, to the field of amyloidosis? You have dedicated several decades to this disease, and you were totally focussed; you built a world-famous institute in Pavia, the Amyloid Research and Treatment Center, dedicated to this very rare entity.

GM: It is a very important question but actually, I must confess that I was somewhat forced to enter the amyloidosis field. I was initiated to the then-mysterious condition of amyloidosis by my two mentors, Jan Waldenström and Elliott Osserman. By the way, I decided to stay with Waldenström because I read his book Monoclonal and Polyclonal Gammopathies which is fascinating as it is full of case presentations with many questions so after staying with Waldenström, he introduced me to Elliott Osserman at Columbia University in New York. Osserman was a world-leader in plasma cell dyscrasia. He had worked before in amyloidosis, and he was the President of the IV Symposium on Amyloidosis in New York, in 1984. So, he more or less conscripted me, despite my reluctance, because at that time the field was so foggy, and nothing was clear. So, we reached a deal that I would work for one year and if nothing came up, I would return to my beloved myeloma proteins. At the Symposium I reported that the main constituent of amyloid fibrils were light chain fragments highlighting the importance of the remodelling in amyloid formation. That gave me the idea that amyloid biochemistry played a key role and that ignited my interest in this disease. This observation was really the trigger of my interest. At the same conference in 1984, George Glenner reported the discovery of the beta amyloid protein forming cerebrovascular amyloid in Alzheimer’s disease. So, I thought that this field was extremely interesting from the biological point of view. Glenner predicted that the understanding of the different molecular mechanisms for amyloid diseases would trigger the development of targeted therapies. I was fascinated by this concept, and we are now finally approaching his goal.

MM: I agree with you, really fascinating. I looked recently at your PubMed publications and found almost 600 papers that you authored or co-authored dedicated to amyloidosis. The total number of citations is almost 40,000; the h-index is 92, but can you tell us, please, what the status of the research on amyloidosis was in the early 80 s when you started, because when I was a medical student, this disease was considered a desperate situation, and nothing worked.

GM: Exactly, amyloidosis was in a dark time for knowledge with foggy pathogenesis and poor recognition, because it was not recognized. I knew hematologists who had not seen any amyloid patients during the length of their career. The lack of therapy meant that, as you said, the diagnosis was a curse with a median survival of 6–8 months. Very little was known about the molecular mechanism of the disease which at that time was grossly divided between primary and secondary amyloidosis. Furthermore, at that time, and you must think about this, studies failed to show the advantage of chemotherapy. It was just a desert. A randomized trial of superiority of melphalan and prednisone compared with colchicine was published by the Mayo Clinic in 1997, and so the field was clearly devastated, and nothing worked because we did not know the disease. There was a confusion over the cause and furthermore there was no patient stratification. The diagnosis was made very late and practically nothing worked. So, I understood that the late diagnosis was the cause of the problem.

MM: So, you started in this field where nothing worked, and in addition to your remarkable training in Italy and at prestigious institutions, you also went abroad to Sweden, and to Columbia University, where you spent a lot of time. Despite all of this you chose to go back to your home city of Pavia. What was the situation of the research, either basic or clinical, on amyloidosis at the University of Pavia when you returned there from Columbia University in 1985.

GM: I will try to explain. When I returned to Pavia there was a desert with no basic research. No clinical research on amyloidosis was ongoing. The disease was rarely spotted and was left untreated and so one of the first initiatives taken was to build from scratch, a laboratory dedicated to protein biochemistry. The second was to improve the awareness of the disease and to this end I established the first network ever in the world, a network of centers to gather patients for clinical trials. The other problem is to reach a critical number of patients to perform trials. It was clear to me that to improve the care of this disease, you need a network, this was vital. This was a very lengthy and stressful process but formed the basis for patient referral to the Amyloidosis Research and Treatment Centre in Pavia. People asked me, why did I ask the hospital manager to give me space for a laboratory? Why did I need a lab? I replied that if I do not do research, I would betray my patients because we need to understand the disease better in order to make progress.

MM: So, you have built everything from scratch in an environment where this rare disease almost did not exist, totally neglected like many other rare and orphan diseases. You mentioned the network and I wonder whether there were people who were active in research at that time that you would like to comment on and who may have influenced you and maybe contributed to creating this positive environment for amyloidosis?

GM: Well, certainly Jan Waldenström played a central role because he introduced me to the concept of ‘sick molecules and diseases’. Firstly, he ignited my interest in the investigation of the pathological effect of monoclonal proteins. Secondly, I learned the importance of very careful patient examination in searching for the molecular basis of the clinical manifestations. The personal discovery by Jan Waldenström came from the observation of a patient, which I will come back to, that was critical for me. In New York, I had the great fortune to work closely with Elvin Kabat, the leading world expert in immunochemistry, a pupil of Michael Heidelberger, often regarded as the father of modern immunology. I learned from Elvin Kabat the rigorous experimental approach and the importance of openly discussing the data with colleagues. Elliott Osserman taught me the importance of translating advances made in the laboratory to the clinic, but most importantly, to discuss and meet colleagues involved in patient research and clinical research in different institutions, such as the Mayo Clinic and Boston University groups in the United-States, and in Europe, the group of Professor Pepys, the Porto group led by Salazar I believe I am deeply in debt to all these colleagues for exchanging ideas for research and therapy. The masters were important but so were colleagues for developing new research avenues. I strongly believe, particularly for rare diseases, that getting together, and networking is vital. That was very important for me as well.

MM: We knew this but when I hear it again, you had remarkable energy to bring people together and you mentioned building this very strong network around a rare orphan disease. You mentioned your masters, but you too became one very quickly and managed to train dozens of amyloidosis doctors who are practicing and helping patients everywhere today. I wonder, how did you manage to attract these young physicians to be interested in such a rare and difficult disease?

GM: A very good question! I believe that enthusiasm is a big driver and when people see you so enthusiastic and motivated, they get motivated and become curious about what you are doing. I always dedicated a lot of attention to the clinic, and to the build-up of a strong local research group while helping the birth of amyloidosis centers worldwide. Many doctors working in centers in Europe, Uruguay, Argentina, or Japan, trained with me, and I was able to found the International Society of Amyloidosis. I think that it is vitally important for collaboration and for improving the care of patients with this now curable disease.

At the beginning of my activity in Pavia, I recruited young people and at the time I was collaborating with the biochemists led by Vittorio Bellotti. Gradually, I selected a motivated basic clinical researcher and together we have built the Pavia Amyloidosis Research and Treatment Centre. Many young people have become important collaborators who have contributed to the growth of the center, not only in Pavia but internationally. I cannot name all of them of course, but the senior researcher Giovanni Palladini, who now directs the center and succeeded me, played a key role and so for me it was a joy to see these pupils grow and become famous internationally and continue the work that I started. I consider this to be one of the main achievements.

MM: I think in every amyloidosis center across the globe, we speak about the contributions of Dr. Merlini. We speak about the way you have built the lab and the way you have built the team. You said that you were very loyal to the teaching of your master, Jan Waldenström, about focussing on the patient. I would like to come back to this relationship with the patient because you are among the very few doctors and colleagues where we know that we can contact you any time of the day or night, and I’ve done it myself several times if you remember, and you would always very kindly answer and give your opinion and try to help. So, what is this very unique relationship you have with your patients, how do you do it?

GM: It is so good to have this conversation with you because you have touched my deep interest. I keep saying to my pupils, we do everything for patients, and we must never forget the core business, the patient. When you forget this, you get lost. The patients have for me been the essential source of inspiration throughout my career. Even when I was very well established, I never skipped the outpatient clinic and I made sure that I could see all the patients, which was in my own interest because you learn constantly from patients, and this was probably the most important thing that I inherited from Jan Waldenström. For instance, in my case, in the early days, conservation of the rapid improvement of the clinical condition was observed after only two months of successful chemotherapy in a patient, that was incompatible with amyloid disappearance that required several months. These kept repeating and this of course, opened up my mind to a new way of thinking, that the amyloid light chain was directly toxic to certain organs. This was corroborated by the finding that concomitant reduction of the concentration of amyloid light chains and of the cardiac biomarker NT-proBNP that we discovered in Pavia. So, this was a case of reversed translational research, from clinic to the lab! This finding had important implications both for basic research and for the care of the patient with amyloidosis because now, we all recognize that it is critical to achieve rapid and deep reduction of the light chains in order to improve the cardiac function and extend survival. Patients have always been my main teachers.

MM: Yes, and thousands and thousands of them across the globe are very grateful to you. Let me move now to a more personal question Dr. Merlini. A few years ago, I met your lovely wife, I do not know about the rest of your family so maybe you could say a few words about the contribution of your wife and family during this exciting career. You dedicated almost all of your time to amyloidosis so what was the opinion of your family?

GM: I would not have done anything without my wife and family. My wife is a splendid person who, in fact, has dedicated her life to me and the family. She has always been here supporting my work and also my two daughters, Arianna and Alessandra who are both doctors, Arianna is a neuroscientist doing research in Göttinger. Alessandra is an oncologist and following in her father’s footsteps by devoting her research into an impossible disease, sarcoma, even tougher than amyloidosis. I would say that all of them are really essential in supporting me. I remember when Alessandra was 2 years old, and my wife asked where is Dad? And she answered, he’s in the laboratory! I try to spend some quality time with them of course but my wife is the center of the family, and they could not have done what they did without her. Another important person is my sister, who has always supported me, even now, so as you said, family has played a key role. Most of the time, I left for the hospital before 8 a.m. and returned at 9 p.m. The only free time I had with the family was Saturday morning, and Wednesday afternoon was for revision of patient records. When I asked my first daughter who had decided to become a doctor, more or less against my will because I wanted her to have a lighter life, she said that I was so happy and enthusiastic in my work so she would like to be like that too.

MM: If you allow me to summarise in a few words, it seems like passion, dedication, commitment, but also sacrifice, is in the genes and DNA of the Merlini family!

GM: That is very generous of you. I think that is very common, seeing you and other colleagues I think that is a common feeling in medicine. I feel privileged doing careful work for other people and that is a common feeling for all of us.

MM: You are a giant, you are a master, and lastly, I would like to ask you one favour. What are your hopes for the future of hematology and most importantly, what is your advice for the younger generation involved in this field today?

GM: Well, Dr Mohty, this is a difficult question, and I do not know if I can answer properly but I believe, for the hematology field, the advent of precision medicine, artificial intelligence and machine learning, and the development of new targeted and effective and less toxic therapies are really revolutionising hematology. I believe that now there is a concrete hope for a cure for many hematological malignancies. Your own work in myeloma and your writing of that splendid paper on Operational Cure in Multiple Myeloma published in *Blood,* testifies that cure is a reachable goal, thanks to all of these. I would like to add a small thought to the fact that the availability of several medical advances, poses a vital question of affordability and sensibility. I am very much involved with these and believe, as a doctor one should play an active role in this complex and multifaceted but critical process. So, I believe we should be clearly informed so that as many patients as possible can realise the wonderful possibilities that we can offer. To the new generation, well, the simplest thing I can say is that there has never been a better time to become a hematologist, and now, more than ever before, it is critical to be a medical scientist. I strongly believe that all doctors in the future will be involved in research because that is really the way to give back to our patients their trust and their hope. Creating this new class of medical scientists is I believe, our responsibility and is going to change the perspective of our patients. The possibility of curing a disease that just a few years ago, was simply considered fatal is the greatest achievement we can hope for.

MM: Well, Dr. Merlini, after hearing this interview, the only words that come to mind are a big thank you, in big capital letters. We owe you a lot, and your energy and passion are incredible, unbelievable, despite your medical condition you manage, and you kindly accepted to give this interview, which I believe is not only a unique moment for me but for our colleagues who are listening to us and watching this interview. On behalf of the medical community, on behalf of the patients’ families and on behalf of future generations, I would like to convey our gratitude and to tell you how privileged we are to have a giant like yourself in our field. Thank you very much.

GM: Thank you Dr. Mohty and thank you to the magnificent scientific community that is accepting me and my work. It has really been a privilege to be part of this incredible adventure. Thank you so much for this opportunity, I am deeply grateful to you and your colleagues.

MM: Please take care and keep well.

## Data Availability

Not applicable.

